# Retroperitoneal Extension of a Fistula-in-Ano: A Case Report

**DOI:** 10.7759/cureus.64240

**Published:** 2024-07-10

**Authors:** Anuradha S Dnyanmote, Prashanth I AM, Rushi Kanani, Hima Shree, Ankita Pandey, Saikumar Immadi

**Affiliations:** 1 Surgery, Dr. D. Y. Patil Medical College, Hospital and Research Centre, Pune, IND; 2 General Surgery, Dr. D. Y. Patil Medical College, Hospital and Research Centre, Pune, IND; 3 Radiodiagnosis, Dr. D. Y. Patil Medical College, Hospital and Research Centre, Pune, IND

**Keywords:** fistula-in-ano, chronic peri-anal fistula, minimally invasive surgical procedures, supralevator abscess, retroperitoneal abscess

## Abstract

Fistula-in-ano and anorectal abscesses are commonly encountered in surgical departments, but their extension into the retroperitoneum and pelvis to form an extensive collection is rare. Here, we present the case of a 66-year-old diabetic male who presented with lower abdominal pain and fever for a day, with signs of sepsis. He had a simple fistula in the perianal region for the past 15 years. Radiological studies showed that the fistulous tract was complex which extended superiorly into the supralevator space and the retroperitoneum and formed a localized collection in the pelvis. The dependent part of the collection was drained using minimally invasive techniques, and the remnant collection was surgically drained through a perianal approach. The patient’s condition improved with further treatment and local wound care, and he was subsequently discharged.

## Introduction

Retroperitoneal abscesses resulting from perianal abscesses are infrequent in surgical practice. Unlike the intraperitoneal region, the retroperitoneum typically mounts a subdued response to bacterial contamination. As a result, retroperitoneal abscesses often advance silently, exhibiting minimal symptoms and progressing slowly over time. This chronic and asymptomatic course presents significant challenges in both diagnosis and management [1]. Delayed diagnosis and inadequate drainage can markedly elevate the risk of mortality and sepsis. Despite treatment endeavors, mortality rates range from 11% to 20% among patients diagnosed with this condition [2].

Multiple factors contribute to the formation of retroperitoneal abscesses. These include perforations caused by colorectal cancers, diverticulitis, retroperitoneal appendicitis, pancreatitis, pancreatic cancer, inflammatory bowel diseases, urinary tract obstruction, osteomyelitis, perforations from postoperative duodenal ulcers, pelvic and postpartum infections, trauma, and the dissemination of infection via the bloodstream or lymphatic system from distant sites. Conditions such as diabetes mellitus, chronic alcohol consumption, glucocorticoid use, malignancies, and distant infections can elevate the risk of complications by impairing the host immune response [1,2]. In our case, we encountered a rare scenario of the existence of the connection between the fistula-in-ano and the multiple retroperitoneal suppurative collections which was a diagnostic, clinical, and surgical challenge.

## Case presentation

**Figure 1:**
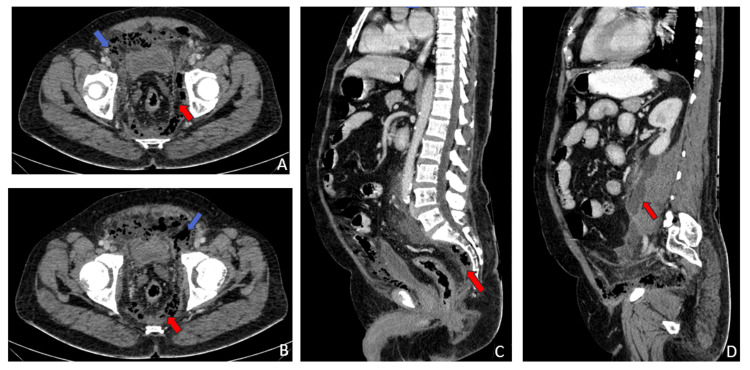
(A, B) Axial CECT of the abdomen and pelvis showing hypodense peripherally enhancing collections with multiple air foci in the presacral region (red arrows) in continuum with similar collections in the perivesical region and underneath the anterior abdominal wall (blue arrows). (C, D) Sagittal reformatted CECT images showing collection with air foci in the presacral region (C) with superior retroperitoneal extension and involving the left psoas muscle (D). CECT: contrast-enhanced computed tomography

A 66-year-old man arrived at the emergency room with complaints of lower abdominal pain, fever, nausea, and vomiting. He exhibited signs of sepsis, including elevated heart rate, low blood pressure, and rapid breathing. He was a known case of diabetes and was on medication. Physical examination revealed tenderness in the lower abdomen, and a simple fistula was noted during a rectal examination. The patient reported intermittent pustular discharge from the perianal region for the past 15 years. The blood picture showed anemia and leucocytopenia. A CT scan of the abdomen showed heterogeneous collections with multiple air pockets in the pelvis and retroperitoneal space (Figures 1A-1D).

**Figure 2:**
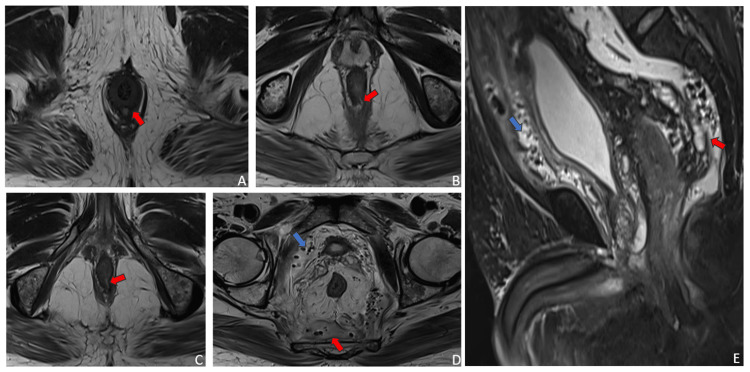
(A) MRI axial T2WI showing the external opening of the fistula at the 6 o’clock position appearing hyperintense on T2. (B, C) MRI axial T2WI showing the ramifications of the fistulous tract opening into the presacral region. (D) MRI axial T2WI showing heterogeneously hyperintense collections with hypointense foci of air in the presacral region (red arrow) and perivesical region anteriorly (blue arrow). (E) MRI STIR sagittal image showing walled-off presacral collection with air foci extending superiorly into the retroperitoneum and anteriorly in the perivesical region (red arrow). MRI: magnetic resonance imaging; T2WI: T2-weighted image; STIR: short tau inversion recovery

Believed to be the source of the sepsis, an emergency ultrasound-guided pig-tailing was performed to drain 200 cc of pus. The patient’s condition showed improvement. Considering the 15-year history of a fistula, the patient was investigated with an MRI fistulogram which revealed a complex fistulous tract extended superiorly to a collection in the ischiorectal space, then into the presacral retroperitoneal space up to the third lumbar vertebra, and tracked along the pelvis and prevesical space beneath the abdominal wall (Figures 2A-2E).

**Figure 3:**
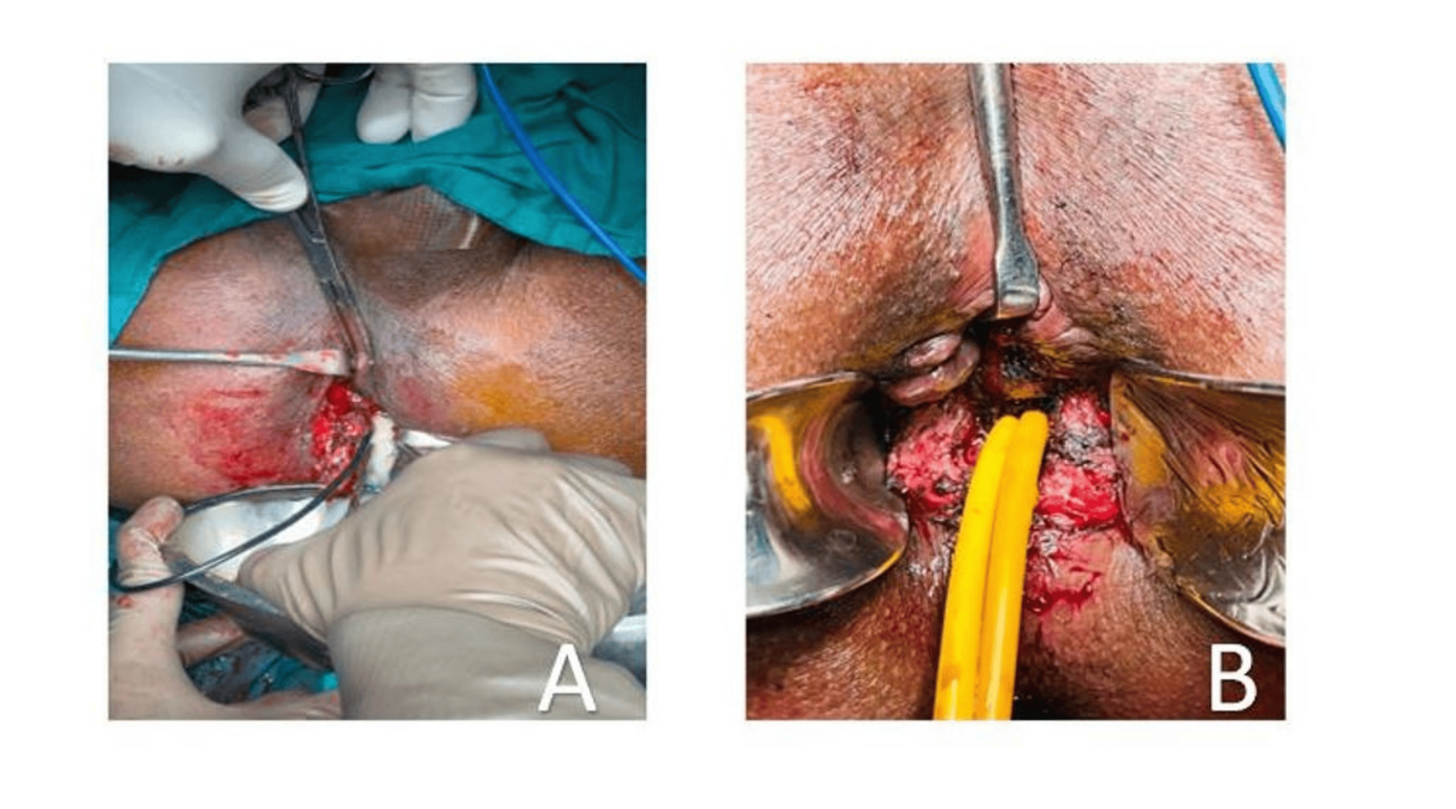
(A) Fistulectomy with drainage of the high supralevator abscess. (B) Postoperatively, a catheter placed in the supralevator space for irrigation and wound care.

The pigtail actively drained the intra-abdominal and the retroperitoneal collection which was dependent on the drain whereas the non-dependent area was not approachable by minimally invasive techniques. Hence, surgery was planned, during which the fistulous tract was removed, and the localized collection was drained from the supralevator space using a fair incision in the perianal region exposing the sphincters and draining the supralevator abscess. A Foley catheter was left in situ to flush the wound during regular dressings (Figures 3A, 3B).

Following surgery, the patient’s condition showed significant improvement, and he was treated with broad-spectrum antibiotics. Culture-specific antibiotics were identified and administered accordingly. The wound received local care under aseptic precautions for one month, and the pigtail catheter in the abdomen was removed after a repeat ultrasound confirmed no residual collection. The patient’s general condition improved, and he was discharged with instructions for regular dressings and follow-up appointments.

## Discussion

Anorectal abscesses are a common surgical concern encountered in everyday medical practice, primarily arising from non-specific cryptoglandular infections. Less frequent causes include conditions such as Crohn’s disease and hidradenitis suppurativa. Depending on their site, anorectal abscesses are categorized into perianal, ischiorectal, intersphincteric, and supralevator types. Perianal abscesses constitute about 60% of all anorectal abscesses. They typically originate from infections in the perianal glands within the intersphincteric space and can extend upwards to form a supralevator abscess. Symptoms commonly involve perianal pain and fever. Patients with diabetes or compromised immune systems are particularly vulnerable. Complications associated with anorectal abscesses include external rupture through the perianal skin or internal rupture into the anal canal, potentially leading to the formation of a fistula-in-ano. However, in immunocompromised patients, clinical presentation may deviate from the typical pattern, with abscesses occurring in atypical anatomical locations [3].

Inflammatory conditions affecting the rectum and surrounding areas are common issues, typically located below the puborectalis muscle. Without prompt diagnosis and appropriate treatment, it is expected that more than 90% of these collections will rupture. The puborectalis sling exerts significant pressure on the posterior rectal wall at the anorectal junction, typically preventing abscess extension above this level into the supralevator spaces. Instead, abscesses often rupture through the longitudinal muscle and spread trans-sphincterically into one of the infralevator anorectal spaces. However, in rare instances, a low intersphincteric abscess may extend above the puborectalis muscle to form a high intersphincteric abscess, which can rupture into the supralevator spaces. Supralevator abscesses can spread into the prevesical space due to direct communication from the pararectal space, where the umbilicovesical fascia terminates at the reflection of the vesical peritoneum. From the prevesical space, infection may disseminate anteriorly throughout the Retzius space and into other pelvic compartments, or posteriorly into the retroperitoneum through direct spread. The retroperitoneum typically responds less vigorously to bacterial contamination compared to the intraperitoneal region, often leading to a more subtle and asymptomatic course. This may result in delayed detection and treatment, potentially increasing the risk of sepsis and mortality [4].

A clinician can only partially assess the retroperitoneum through physical examination, and laboratory tests provide limited information. Therefore, radiological investigations are crucial for accurate diagnosis. CT is particularly valuable in evaluating the retroperitoneal region. Additionally, MRI has demonstrated effectiveness in assessing the relationship of complex horseshoe abscesses with the deep postanal region, as well as in evaluating secondary abscess formations, additional fistulas, internal openings, the extent of abscess spread, and sphincter involvement [5,6]. In our case, MRI played a vital role in diagnosis. Deep-spreading perianal abscesses are considered severe in anorectal disease due to their rare occurrence and subtle clinical symptoms, which may lead to delayed diagnosis, severe sepsis, and potentially fatal outcomes. Conditions such as inflammatory bowel disease, heart disease, obesity, or diabetes mellitus increase the risk of complications [7]. The absence of typical signs alongside abdominal pain can complicate diagnosis for physicians evaluating abdominal pathology. Retroperitoneal abscesses can stem from the genitourinary tract, be idiopathic, postoperative, or occasionally involve other organs such as the colon, duodenum, and pancreas. Typically, treatment involves lumbar incisions, as the transperitoneal approach is less effective. The mortality rate can reach 26%, especially among critically ill patients with delayed diagnoses [8].

Treatment approaches are diverse, typically favoring open drainage and debridement as the established norm. Managing limited supralevator abscesses presents challenges in percutaneous intersphincteric drainage, where fluoroscopic monitoring may prove insufficient, potentially leading to prolonged sepsis [9]. When dealing with extensive pre- or retroperitoneal extension, it is advisable to avoid accessing the peritoneal cavity to reduce the risk of contamination and secondary peritonitis. Effective treatments include drainage by abdominal incisions or performing extraperitoneal drainage through lower midline abdominal incisions, typically closed primarily with drains or vacuum-assisted devices [10,11]. Complex cases described in the literature, such as ischiorectal horseshoe abscesses with anterolateral extraperitoneal spread, have been effectively treated with comprehensive surgical interventions. These include open drainage and canalization through the ischioanal fossa via abdominal incision [12], and simultaneous fistulotomy. On the other hand, difficult cases where ileus is caused by an enlarging abscess have been managed successfully with direct drainage following a challenging diagnosis [13,14].

In the past, surgery served as the mainstay treatment for retroperitoneal abscesses until the advent of imaging-guided percutaneous drainage offered an alternative. Initially used for uncomplicated abscesses, percutaneous drainage has advanced to handle complex cases involving multiple compartments or fistulas in regions such as the pancreas, spleen, or retroperitoneum [15]. This minimally invasive approach has demonstrated significant advantages in lowering mortality and complications, particularly in high-risk patients or those with prior surgical history, compared to conventional surgical techniques [16,17].

## Conclusions

This case report highlights the rare challenges encountered in surgical practice when dealing with perianal issues, emphasizing the complexities that challenge surgeons in various aspects. A thorough understanding of the perianal and retroperitoneal regions is crucial for surgeons to make informed decisions regarding surgical management. Surgical drainage of suprarectal abscesses is pivotal in patient care, and minimally invasive techniques are increasingly important for managing retroperitoneal and intraperitoneal abscesses. A multidisciplinary approach leveraging advancements in surgery and radiodiagnosis plays a significant role both before and after surgery in managing such complex cases effectively.
